# Clozapine placental passage at delivery: an update

**DOI:** 10.1192/j.eurpsy.2022.610

**Published:** 2022-09-01

**Authors:** M.L. Imaz, S. Lera, E. Roda, A. Roca, A. Torres, E. Solé, S. Andres, A. Mallorquí, L. Garcia-Esteve

**Affiliations:** Hospital Clinic, Unit Of Perinatal Mental Health Clínic-bcn. Department Of Psychiatry And Psychology, Barcelona, Spain

**Keywords:** Delivery, Mother-infant pair, Placental passage, clozapine

## Abstract

**Introduction:**

Clozapine is an effective second-generation antipsychotic that is approved for treatment-resistant schizophrenia and risk reduction of recurrent suicidal behavior in schizophrenia or schizoaffective disorder. Its available pregnancy pharmacikinetics data remain limited, which presents a challenge for clinicians managing women taking clozapine during perinatal period .

**Objectives:**

The aim of this study was to provide new data of clozapine and norclozapine placental passage and neonatal outcomes.

**Methods:**

We retrospectively studied a consecutive case series of six pregnancies where there was clozapine exposure (5 in politherapy and 1 in monotherapy). Clozapine and norclozapine serum concentrations were determined in the mother-infant pairs on the day of delivery (intrapartum maternal blood and umbilical cord blood respectively) and measured using a validated high-performance liquid chromatography method. The within- and between-day precision expressed as the coefficient of variation (CV)% were both <10%. The limit of quantification (LoQ) was 5 ng/mL. Neonatal outcomes were reviewed from pediatric records.

**Results:**

The mean infant-mother clozapine and norclozapine ratio at delivery were 0.44 (SD=0.13) and 0.28 (SD=0.05) respectively. There was a weak positive correlation between maternal and umbilical cord clozapine and norclozapine serum concentratios (Pearson correlation coefficient 0.183, p=0.769 and 0.827, p=0.084 respectively). The rate of neonatal complications was 16%. One neonate (16%) , whose mother had drug abuse history during pregnancy, presented with a generalized neurodevelopment delay and the consequent need for continuous intensive care.

**Conclusions:**

In our study, placental passage of clozapine and norclozapine was partial during delivery. Statistical power was limited for examining te association between neonatal clozapine levels and neonatal outcomes.

**Disclosure:**

No significant relationships.

